# The Design and Implementation in Python of Aspects of a Service Orchestration Engine for a Smart Port Ecosystem

**DOI:** 10.3390/s26216907

**Published:** 2026-10-31

**Authors:** Bibin Babu, Cathryn Peoples, Joseph Rafferty, Adrian Moore, Nektarios Georgalas

**Affiliations:** 1 Faculty of Computing, Engineering and the Built Environment, Ulster University, York Street, Belfast BT15 1ED, UK; b.babu@ulster.ac.uk (B.B.); j.rafferty@ulster.ac.uk (J.R.); aa.moore@ulster.ac.uk (A.M.); 2 Research and Network Strategy, BT, Martlesham IP5 3RE, UK; nektarios.georgalas@bt.com

**Keywords:** Internet of Things (IoT), optimization, orchestration, Python, quality of service (QoS), simulation

## Abstract

The Internet of Things (IoT) continues to expand its reach. To support the interconnection between moving entities, the Internet of Moving Things (IoMT) has emerged. As IoMT and IoT applications continue to expand in their capability, it is crucial to ensure that services are reliable and efficient, which is where Service Level Agreement (SLA) fulfillment comes into play. An Orchestrator can support the achievement of the SLA made available between customers and a network provider and help to avoid any circumstances of compensation needing to be paid—the Orchestrator is a critical block in an IoT ecosystem, which coordinates and manages devices, sensors and actuators, network infrastructures, services and applications. When a customer moves around within an ecosystem, both the customer and ecosystem may need to adapt to the real-time conditions for continued fulfillment of the SLA. In emergency situations, prioritizing human safety takes precedence, potentially diverting resources away from maintaining agreed-upon service levels. The challenge of this is further compounded by the fact that there are multiple stakeholders within a domain, such as end-users, network operators, service providers, and regulatory bodies, each of which have varying network demands. In this paper, the design and implementation of aspects of an IoT Orchestrator supporting SLA achievement are discussed, with a port ecosystem used as a case study. We demonstrate the impact of the Orchestrator in several simulated maritime port use case scenarios, utilizing a custom-built simulator to evaluate the solution’s effectiveness.

## 1. Introduction

The number of devices connected to an Internet of Things (IoT) ecosystem continues to increase. With this growth, the data collection, resource allocation, and management of customers become challenging from the perspective of ensuring that sufficient resources exist to meet demands and that effective decisions are made. An orchestrator of the network resources within the environment can facilitate the making of such decisions and play an important role in ensuring that a Service Level Agreement (SLA) made between a customer and service provider is fulfilled.

When it comes to the case of the Internet of Moving Things (IoMT) in particular, the number of customers within an ecosystem can change significantly over time. Variation in the number of customers introduces challenges such as the dynamic need for resources, change in the volume of data being generated and handled, and adapting to the interests of the customers for which the ecosystem is operated. In the event of an emergency, such as a vehicle exceeding a threshold speed limit for an area, prioritizing human safety takes precedence over general monitoring to prevent potential harm or risk to life. The IoMT ecosystem presents a complex optimization challenge and a multi-objective problem-solving task. The development of an orchestrator that has the capability to address these issues is equally challenging. Current ecosystems are not dominated by a single platform, and different systems exist to address device management, edge computing, interoperability and orchestration. Each prioritizes and focuses on specific aspects of operation, and one is not necessarily a direct substitute for another. This paper details the design and development of such a system, intended to support the interoperability required of an IoMT management tool through exploration of the following research question: How can an interoperable IoMT management system be designed and developed to integrate heterogeneous platforms and services for device management, edge computing, and orchestration within a distributed IoMT ecosystem? This paper details the design and development of such a system.

The main contributions of the paper can be summarized as follows:

- Development of a flexible and scalable IoMT Orchestrator: An Orchestrator can support the effective management of IoMT ecosystems, encompassing resource allocation, device and data management, quality of service (QoS) assurance, and application and service orchestration.
- Evaluation of the Orchestrator’s performance under emergency scenarios: The study investigates how unexpected or critical events, such as emergencies, influence ecosystem behavior and the Orchestrator’s decision-making efficiency.
- Assessment of resilience and adaptability mechanisms: Strategies are explored to ensure that the Orchestrator maintains stable operation and can dynamically recover or adjust to changing IoMT conditions.
- Definition of key performance indicators (KPIs): Specific KPIs are established to quantitatively measure and benchmark the Orchestrator’s performance, forming a foundation for further optimization and research.

A simulation framework is built to generate metadata and model scenarios which replicate the IoMT, allowing the Orchestrator’s overhead and performance to be evaluated.

The remainder of this paper continues as follows. A study on state-of-the-art orchestration engines has been conducted in Section 2. Section 3 provides the list of objectives driving development decisions of the proposed model. A detailed discussion on the development and implementation of the system is given in Section 4. It details the generation of simulation data and orchestrator development. The discussion of results and performance evaluation of the developed model is presented in Section 5. Finally, Section 6 outlines the conclusions and future research potential.

## 2. Literature Review

A variety of architectural models, three of which are discussed below, are considered within the context of IoT orchestrators. These architectures differ in their consideration of system views. The ANSI/IEEE std 42010:2022; for example, describes a solution from the perspective of its architecture, stakeholders, concerns, viewpoints, and correspondences [1]. The concept proposed is intended to support the creation of an Orchestrator but is not the Orchestrator in itself.

The ISO/IEC 30141:2024 Internet of Things (IoT) IoT—Reference Architecture, on the other hand, presents six architectural viewpoints: Foundational, Business, Usage, Functional, Trustworthiness and Construction [2]. It provides a useful architectural foundation for developing an IoT orchestrator, but again, is not the orchestrator in itself. Its value is that it helps to define the orchestrator’s responsibilities and architecture.

The frameworks provide principles, models, viewpoints and rules for describing interoperable systems, which the orchestrator coordinates real operational activities across those systems; it is a software capability.

The TM Forum’s New Generation Operations Systems and Software (ngOSS) architecture shows an organized approach to the design of the system [3,4,5]. It consists of two viewpoints: physical and logical. The physical view addresses implementation and deployment, emphasizing the firmware, hardware and software required for system operation functionality and monitoring. The logical view, on the other hand, focuses on the business and system dimensions, entities, detailing processes, interactions, behaviors, managed objects and computational interactions. The Open Digital Architecture (ODA) by the TM Forum is an evolution from the ngOSS model, adopting adaptability and openness within the IoT ecosystem [6]. It introduces ODA components, which are modular software entities deployable independently; the ODA canvas is an operating environment tailored for Cloud Service Providers (CSPs). With Gen 5 Open APIs, the architecture ensures seamless interoperability with contemporary solutions. Additionally, the System Service Layer allows ODA to integrate with different cloud platforms, enhancing its versatility [7]. This innovation extends to supporting autonomous and sustainable IoT systems, focusing on mobility and efficiency.

We consider a strong relationship to exist between the ngOSS/ODA and the orchestrator, which is proposed as a part of this work, in the sense of the related architectural layers, a standardised telecom architecture, and a solution to coordinate IoT devices, connectivity and services. To contrast, the ODA provides the architectural framework, and the orchestrator performs the actual coordination of IoT resources and services.

Prior work in container-based IoT management has shown how Kubernetes and cloud platforms can provide reliable, maintainable and scalable infrastructures for IoT services, using MQTT for device connectivity and GKE for deployment [8]. These technologies play different but complementary roles in an IoT orchestration system and are therefore relevant to consider here; however, they should be recognised as not being equivalent orchestration technologies: MQTT is a messaging protocol, Kubernetes orchestrates containerised workloads, and GKE is Google’s managed Kubernetes service. Work on big-data orchestration (BDCE) provides a platform-agnostic microservices and GUI-driven approach for constructing reproducible data pipelines and YAML-based workflows but is oriented toward throughput and analytic workflows rather than real-time mobility and safety concerns [9]. Recent cloud-native slicing work (SliceSphere) advances hierarchical, slice-oriented orchestration and marketplace tooling to achieve strong isolation and multi-cloud deployment, yet it is focused on general application slicing rather than IoMT-specific emergency prioritization and per-customer SLA trade-offs [10]. In contrast, the Orchestrator proposed in this paper targets IoMT mobility, formulates multi-objective orchestration (SLA, safety, cost), and evaluates behavior with a dedicated simulator and KPI set—addressing the gap between cloud/container operations, big-data.

A comparative analysis on state-of-the-art orchestration solutions is given in the Table 1. In comparison with other solutions, the proposed model predominantly focuses on IoMT devices, providing high scalability and low latency. Adaptability and maintenance of the SLA in emergency conditions are the key objectives.

## 3. Research Proposal

The aim of the work presented in this paper is to develop an IoT orchestration engine, which is responsible for observing and monitoring the IoT environment, detecting potential situations of resource constraint or need to reoptimize the setup configuration, and enforcing the necessary manipulations in response. The scope of the orchestrator includes service and workload placement, resource allocation, device lifecycle management, and fault handling [11]. The proposed model addresses multiple objective optimization problems from the perspective of different stakeholders and extends upon our prior work, e.g., [12]. Multiple objective optimization means that the Orchestrator must simultaneously fulfill the requirements of all stakeholders, including achievement of the SLA, health of the IoT environment, financial stability of the ecosystem, and environmental safety. In the developed model, we consider a maritime port scenario in which stakeholders include customers moving in and out of the ecosystem, the port authority, logistical transport trucks, and IoT network service providers. A port is a highly dynamic environment that can be considered to operate as a smaller, enclosed smart city. Stakeholders come in and out of the environment, place varying levels of demand on the network, and have high expectations with regard to performance. Furthermore, in this environment, there are humans working and existing here with highly varying priorities—some in a relaxed holiday mode and others in a heavy industrial role. Networking solutions here, however, can be limited in their capability due to their remoteness and lack of specialized expertise. Orchestration can help to create more autonomy with regard to the solutions in a manner personalized to the way in which the network is being used. The environment can operate in different modes by giving priority to different stakeholder needs through assessing the ecosystem’s current state. The main focus is on the Orchestrator’s influence on the ecosystem when an emergency condition arises in terms of the areas and aspects of the ecosystem affected by the emergency condition [13]. The Orchestrator prioritizes safety in this circumstance, ideally without affecting pre-agreed customer SLAs.

**Figure 1:**
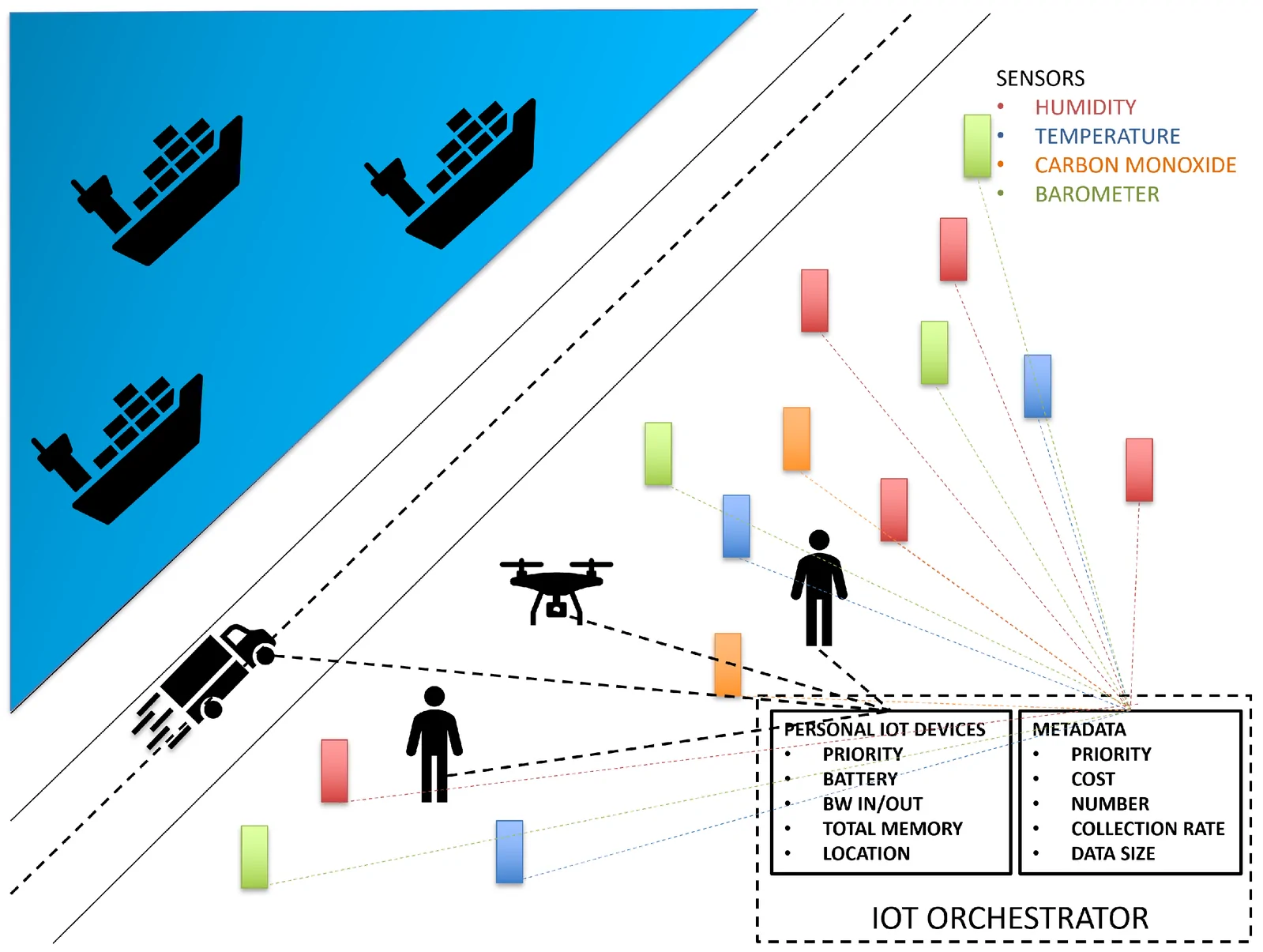
Port scenario setup for the development of the Orchestrator.

The port scenario consists of a number of different sensors, customers, vehicles, and a drone that monitors the environment. To collect different kinds of metadata such as temperature, humidity, atmospheric pressure and carbon monoxide levels, sensors of each type are involved. The drone moves around the port and observes the movement of people, vehicles and ships through its metadata collection, which is subsequently fed back to the Orchestration engine. In the proposed evaluation scenario, a vehicle enters the port and, at a point in time, moves in a way that exceeds an acceptable speed limit. It maintains this above-threshold speed for a period of time before returning to within the speed limit toward the end of the evaluation period. A graphical representation of the scenario is shown in Figure 1.

## 4. Orchestrator Implementation

The Orchestrator continuously monitors environmental events. It actively checks customer status every second to detect new arrivals or departures. Utilizing data collected from various stakeholders, the system assesses potential risk scenarios associated with human life in and around the port. The implementation reported in this paper consists of two stages:

- Generation of the metadata
- Development of the Orchestration system

### 4.1. Generation of the Metadata

Two contrasting scenarios are considered to evaluate the performance of the proposed mechanism. The first scenario involves a vehicle speeding up in the port above an acceptable speed threshold. Two customers are present in this scenario; GPS coordinates and accelerometer values are generated to calculate the vehicle’s speed at each timestamp, continuously for a certain period of time. The second scenario simulates multiple customers entering and exiting the port area, collecting GPS coordinates for each customer and generating coordinates for sensor movements.

#### 4.1.1. Generation of GPS Coordinates for Simulation

In both scenarios, GPS coordinates are the primary collected data. For the scenario where the vehicle speeds up, since it involves two customers and a vehicle, for the generation of GPS coordinates, a primary point is established, and three points at specific distances are calculated. The primary point is considered as the center of the environment, and the other three points are the starting positions of the customers and vehicle movement. Random GPS coordinates are then generated and sorted to simulate movement toward the primary point, resulting in continuous vehicular and people movement. The coordinates for two people are generated from different directions, with proximity to the vehicle decreasing over time. To assess the proposed scenario more efficiently, both vehicles and customers are considered to move towards the center of the environment, ensuring that they remain within the environment, and the proximity between vehicles and customers decreases over time, allowing for evaluation of the effect of emergency conditions. To enhance the realism of the simulation, accelerometer values are generated randomly within a range, and the vehicle’s initial velocity is calculated from GPS coordinates, with subsequent velocities calculated using accelerometer values.

**Figure 2:**
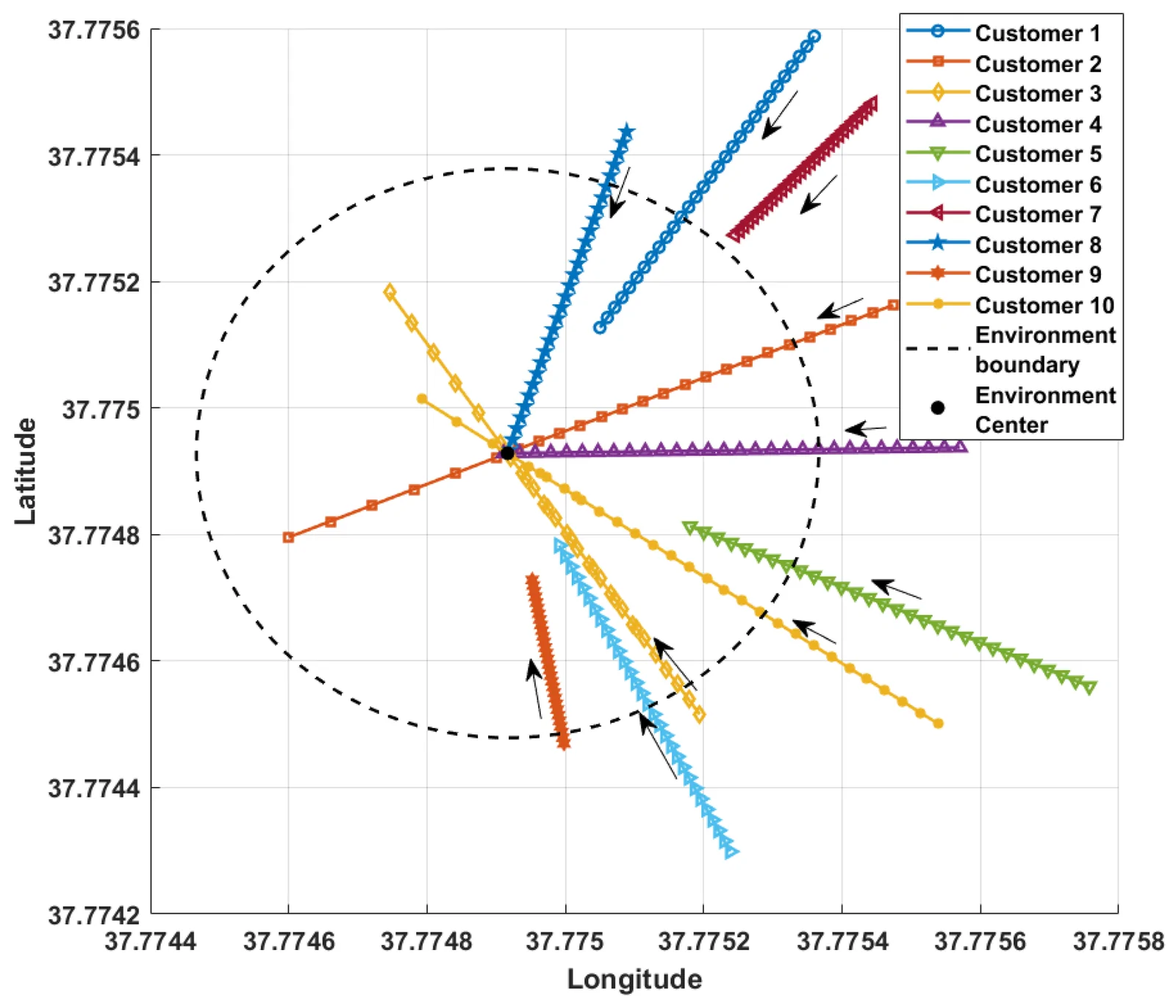
Graphical representation of customer movement using simulated GPS coordinates in the customer entry and exit scenario. The arrows indicate the direction of customer movement.

For customer entry and exit to and from the ecosystem and sensor movement, GPS coordinates are generated. A plot of ten customers’ GPS coordinates generated for 28 s is shown in Figure 2. The proposed model can accommodate an unlimited number of customers; however, for ease of representation, the simulation assumes ten. The simulation runs for 30 s, with the first 2 s used to calculate initial values. Consequently, the graphical representation only displays the remaining 28 s. The black dotted circle in the figure shows the service boundary of the environment. A central point serves as the environment’s hub, and multiple points are calculated for customers and sensors at varying distances and directions. From these points, coordinates are calculated at each timestamp for randomly generated distances within a specific range. As time progresses, customers and sensors initially move towards the center point, then away from it. The distance range ensures that the number of customers within the environment increases and decreases over simulation time, achieved by setting a boundary distance from the center. Customers within the boundary distance at each timestamp are considered inside the environment; otherwise, they are outside. Similarly, the GPS coordinates to represent sensor movement are generated within the boundary distances, without considering the boundary concept. The simulation of GPS coordinates is designed such that customers move towards the center of the point and then move away, continuing on their forward path, facilitating evaluation of the model’s performance. As shown in Figure 2, the number of customers within the environment increases over time. Some customers pass the ecosystem hub, but none exit the environment. Generation of the GPS coordinates is shown in Algorithm 1.
**Algorithm 1:** Generation of Customer Coordinates
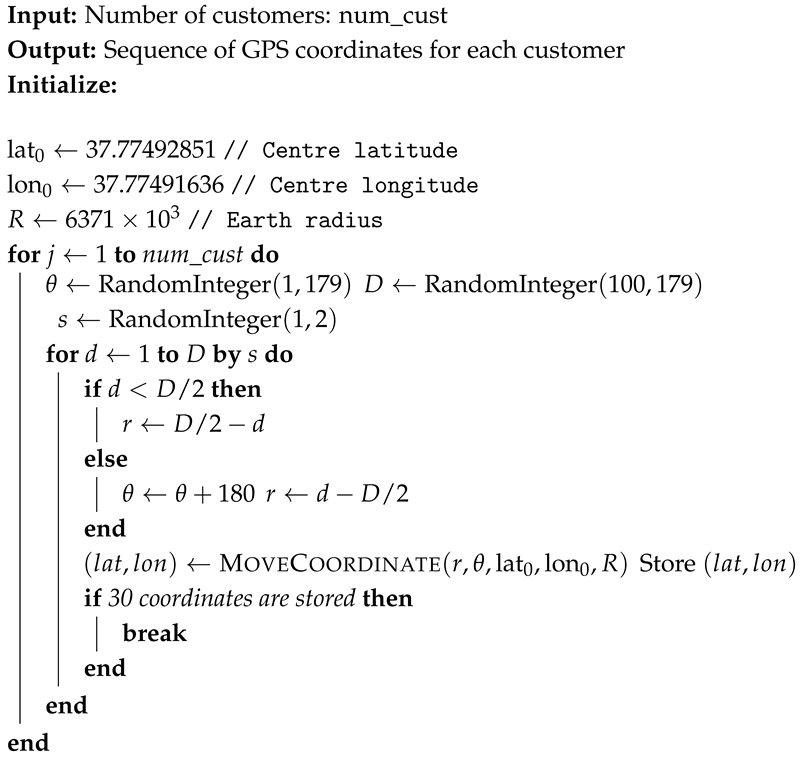


The proposed IoMT orchestration framework defines three SLA plans with different service requirements to support varying application priorities and resource constraints. Each plan specifies requirements related to cost, memory, jitter, latency, uptime, bandwidth, packet throughput, packet loss, CPU utilization, response time, battery level, frame rate, and metadata collection rate. Plan 1 provides the most stringent service requirements, while Plan 2 provides intermediate requirements, and Plan 3 provides more relaxed requirements. For simulation purposes, SLA plans are added to the customers randomly. These SLA parameters are used by the orchestration mechanism to determine the appropriate service level and dynamically manage IoMT resources according to application requirements and system conditions.

#### 4.1.2. Generation of Sensor Battery Values and Vehicle Accelerometer Values

As the simulation focuses on the evaluation of the proposed model, the initial values for sensor batteries are generated randomly within a range. This range helps to assess the impact of battery level on data collection range. Two critical battery levels trigger changes in the data collection rate. Additionally, the rate of battery depletion depends on the data collection rate. Similarly, the vehicle accelerometer values are also generated from a specific range to allow the vehicle to come to a speed above the threshold speed during the simulation before returning to a speed below the threshold speed.

### 4.2. Development of the Orchestration System

The Orchestrator tracks the movement of humans, vehicles, and sensors. Based on these trackings, and by assessing network conditions, several adjustments may be applied to ensure that the SLAs continue to be achieved.

#### 4.2.1. Ecosystem Operational Principles

A customer is considered to exist within the environment if their distance from the center point is less than the boundary distance. The environment operates in three modes, with one mode being applied at any time: financial, environmental, or performance. Each mode prioritizes cost reduction, environmental health, and quality of service, respectively. This has been done by assigning scores to each metadata attribute and calculating the total score for each mode by taking the sum of individual attributes by assigning a weightage based on the preferred mode.

The mode of operation dynamically affects metadata collection rates, which are adjusted based on available bandwidth (BW). Of the maximum available, 80% of the BW is utilized for data collection. The available BW is not fully utilized; 20% of the available BW is reserved as a backup to be utilized in case of SLA violations. Metadata collection rates are calculated according to the allocated BW portions. BW allocation for different metadata types depends on the environment’s mode of operation. Although it is possible to manually trigger a mode of operation, selection of the mode depends on various factors. Specifically, there is a score associated with each mode of operation, which is calculated by the system itself from the factors that are relevant to the mode. The preferred mode of selection and the high weighting for the preferred mode of operation in the calculation of scores for different modes of operation, which include performance, sustainability and cost-effectiveness, are based on the interest of different stakeholders. For example, in the case of the financial mode, cost reduction is the priority. To reduce the cost of operation, the expense of data collection is calculated for every time stamp. Whenever a particular mode is triggered, a higher weightage is added - this weightage comes from the cost of data collection during each time stamp and determines whether the environment needs to move into operating in a financial mode. The weightage is assigned such that during normal ecosystem operation, the preferred mode of operation is selected. However, if an abnormal condition arises, even with increased weightage, the preferred mode will not be chosen.

In the case of an emergency situation, anonymization of collected metadata is turned off and the emergency mode is activated—there is an assumption that all metadata will be anonymized by default. Anonymization means that instead of not being informed of specific detail regarding the scenario, the system prioritizes the criticality of a potentially dangerous situation. In the emergency operation mode, priority is given to safety and it reflects on the data collection range. The sensor data collection rate is reduced, and the frame rate is increased to assess the unsafe condition more efficiently. This is achieved by allocating a large portion of the available BW to the frame rate collection. If there is an unsafe condition, the other modes are deactivated and the emergency mode is activated, irrespective of the scores of other modes. Algorithm 2 shows the selection of the operational mode of the ecosystem. In different modes, the available BW is allocated to data collection in varying proportions based on the relevance of metadata attributes in each mode. For example, in emergency mode, a high proportion of BW is allocated to frame rate, prioritizing safety. After selecting the mode of operation, the domain optimizer calculates the data collection rates according to the available BW. This rate is then compared with the minimum acceptable collection rate specified in the SLAs of current customers in the environment, and the higher rate is chosen.The performance caps, which will be discussed in the following sections, have been selected to be representative of benchmarks, which will support demonstration of the orchestrator’s capabilities within the context of this simulation.
**Algorithm 2:** The mode of operation of the eco-system
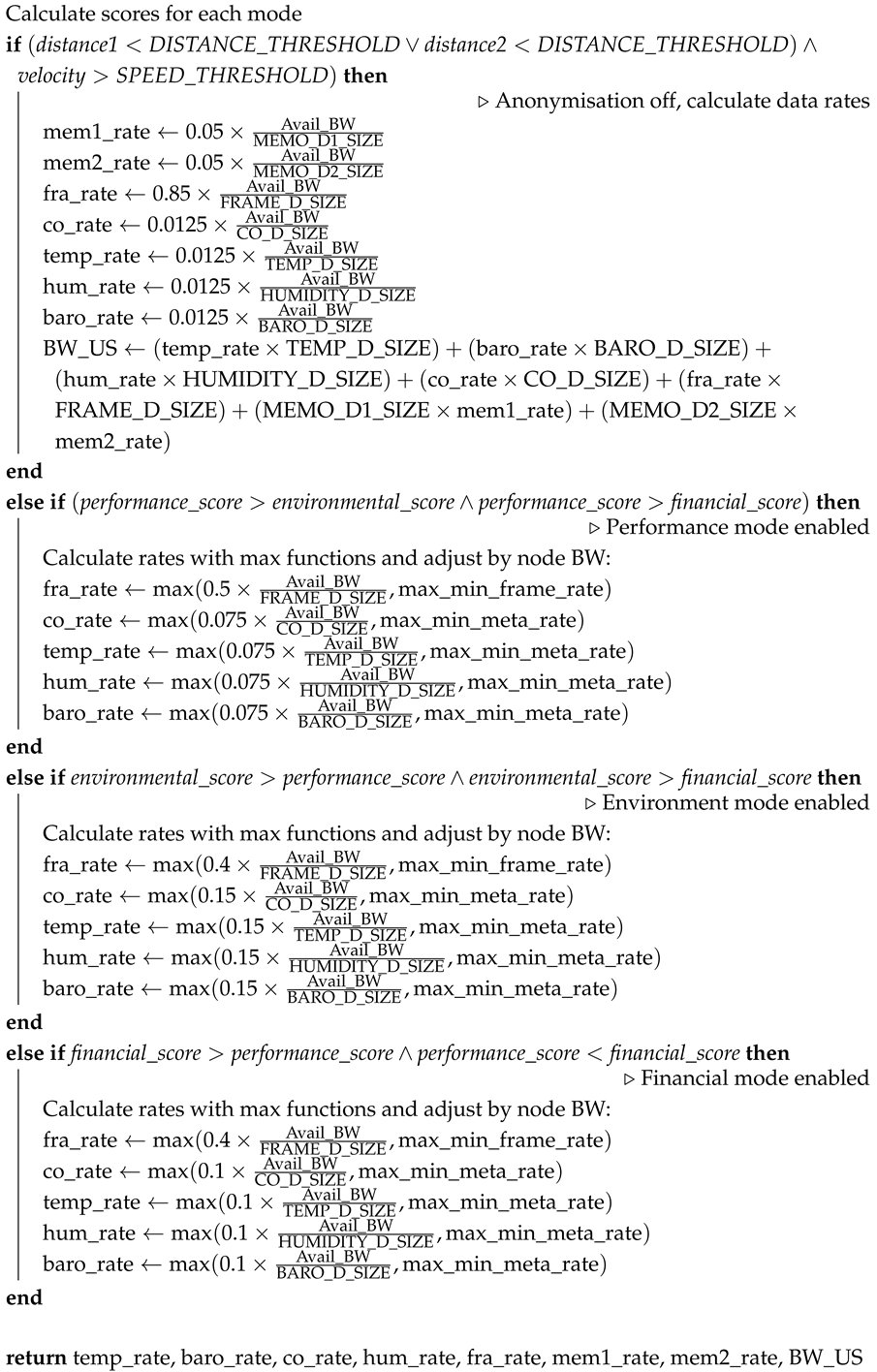


#### 4.2.2. Additional BW in the Case of High BW Usage

**Figure 3:**
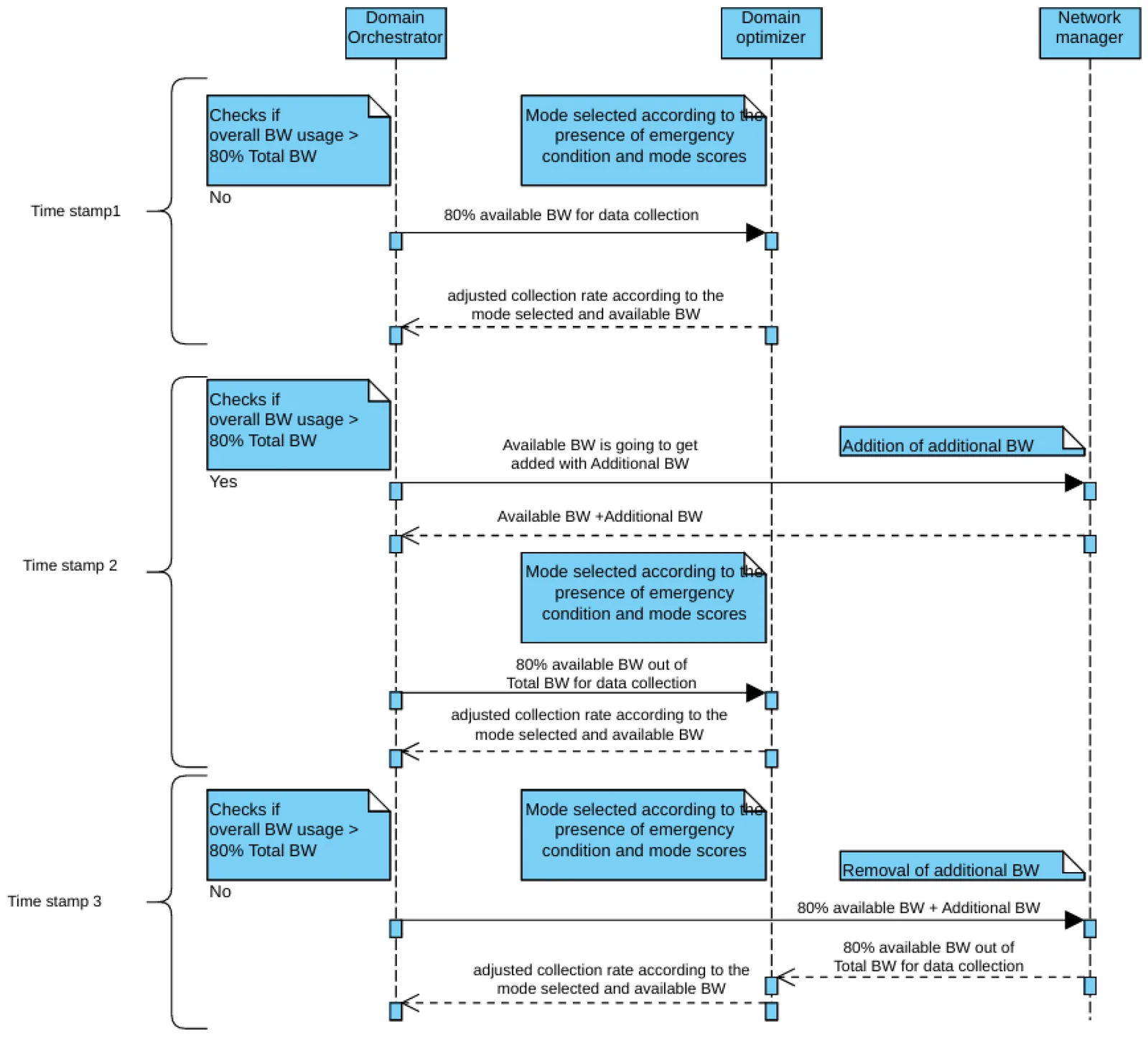
Addition and Removal of Additional BW in the case of High BW Usage.

80% of the available BW is utilized for data collection. BW usage is checked every timestamp by the Orchestrator. If the BW usage based on the BW available is greater than 80%, additional BW is provided by the network manager. Additional BW added is not utilized for data collection. After the addition of extra BW, on the next timestamp, the system again checks for BW usage. If it is less than 80%, the extra BW provided is removed, otherwise it remains (Figure 3). At timestamp 1, the BW usage is less than 80%, so the 80% of the available BW is used for the data collection. The optimiser module of the Orchestrator returns the updated collection rate according to the mode of ecosystem operation. On timestamp 2, if BW usage is more than 80%, additional BW is made available, and 80% of the system BW is utilized for the data collection. On timestamp 3, since the BW usage is less than 80%, the additional BW is removed.

#### 4.2.3. Cost Associated with Data Collection

The cost of data collection associated with each customer is also calculated for each time stamp, as shown in Algorithm 3, and the notations used are provided in Table 2. The data collection cost associated with each data type depends on the subscription priority and the data collection size and rate. This calculation is not intended for customer billing purposes. As the data collection rate depends on the available BW, it can vary with each time stamp. The data collection rate varies for each attribute, based on the priority of the data.
**Algorithm 3:** Calculate Total Cost per Second$C_{r,T},C_{r,B},C_{r,H},C_{r,CO},C_{r,M},f_{r},P$**Calculate cost per second for each attribute:**$C_{M}\leftarrow \frac{C_{c,M}}{C_{r,M}}\times D_{s,M}\times 10$$C_{T}\leftarrow \frac{C_{c,T}}{C_{r,T}}\times D_{s,T}\times 10$$C_{B}\leftarrow \frac{C_{c,B}}{C_{r,B}}\times D_{s,B}\times 10$$C_{H}\leftarrow \frac{C_{c,H}}{C_{r,H}}\times D_{s,H}\times 10$$C_{CO}\leftarrow \frac{C_{c,CO}}{C_{r,CO}}\times D_{s,CO}\times 10$$C_{F}\leftarrow \frac{C_{c,F}}{f_{r}}\times D_{s,F}\times 10$**Calculate total cost per second based on subscription priority:**$C_{\mathrm{total}}\leftarrow \left(C_{M}+C_{T}+C_{B}+C_{H}+C_{CO}+C_{F}\right)\times P\times 2$**return **$C_{\mathrm{total}}$

#### 4.2.4. Effect of Sensor Battery Values on Data Collection Rates

The sensor battery value decreases at a rate proportional to its data collection rate. When the battery reaches 15%, to decrease the rate of battery drainage, the data collection rate is set to the minimum with a ’battery low’ alert. The minimum collection rate comes in accordance with the customer SLA. When the battery level reaches 5%, the battery is replaced by a fully charged battery and the collection rate returns to the initial default.

#### 4.2.5. Background Script for Orchestrator

An Orchestrator script has been implemented to support background operations of the port ecosystem. Threading capability allows the Orchestrator to achieve its functionality in parallel with the ecosystem operations. The background script employs a moving average algorithm, calculating the average of different attributes such as jitter, latency, number of customers, memory usage, BW usage, frame rate, acceleration of sensors and changes that occurred in the GPS coordinates over a five second sliding window (shown in Algorithm 4). This enables the Orchestrator to update the live performance of the ecosystem. This update facilitates informed decision-making and efficient resource allocation.

The 5 s sliding window was selected to provide sufficient recent observations for reducing short-term fluctuations while retaining the ability to capture changes in the system. The simulation duration is set to 30 s for the experiments presented in this study and can be adjusted according to the required simulation period. The first two seconds are used to obtain the initial parameter values, while the remaining 28 s are used for the subsequent simulation analysis. To maintain the intended temporal progression of the simulation, a 0.5 s execution delay is introduced within the processing loop, ensuring that the loop operates at the required one-second observation interval while allowing sufficient time for the associated computations and data updates. The 28 iterations used in the presented implementation correspond to the selected 28 s simulation analysis period and are not a fixed limitation of the proposed approach. The number of iterations can be increased or decreased according to the required simulation duration.
**Algorithm 4:** Calculate Rolling Averages with Threading
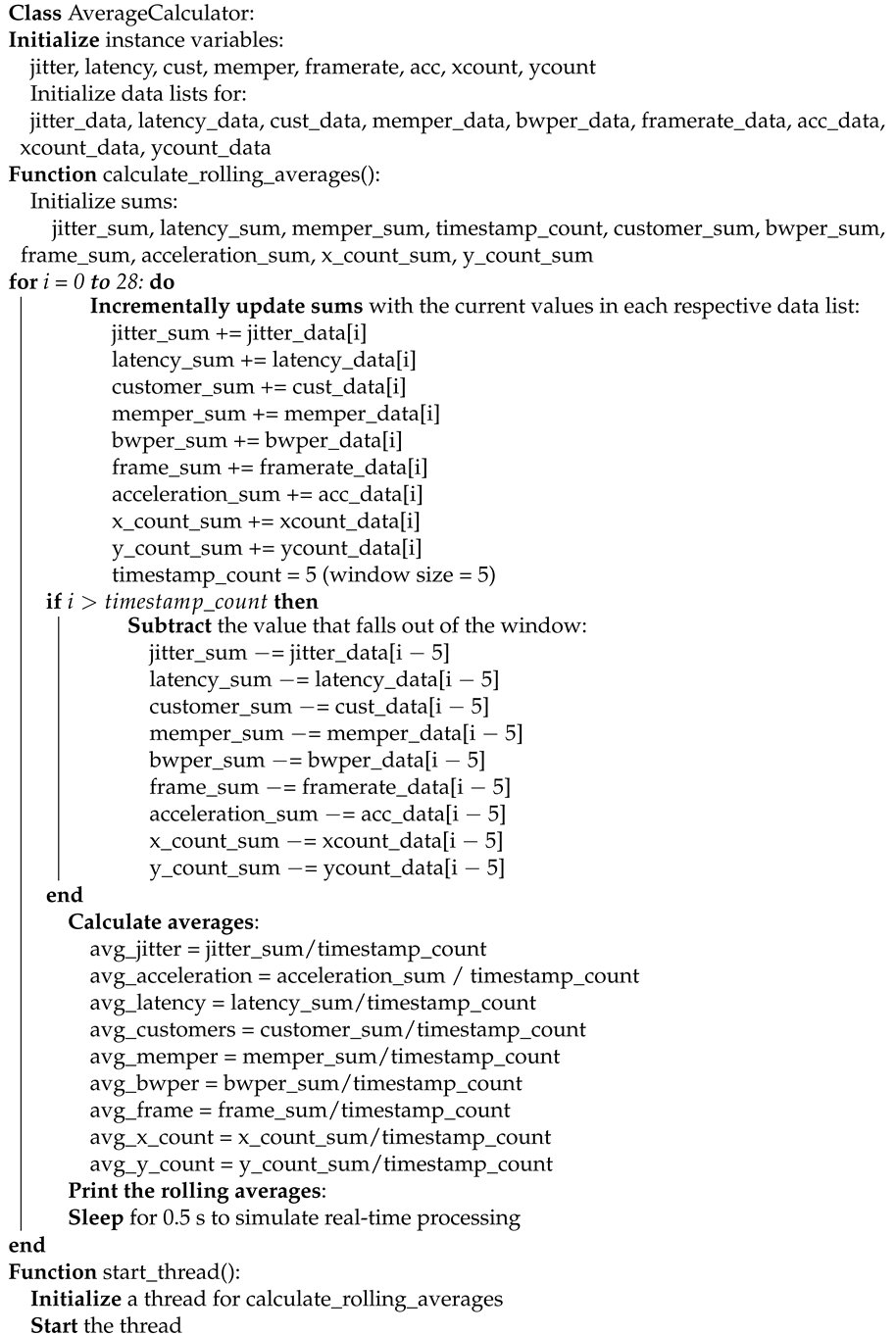


#### 4.2.6. Haversine Distance Calculation of Two Points

Every point in the ecosystem is specified by its GPS coordinates. The distance between two points is calculated from GPS coordinates using a Haversine formula (Algorithm 5). Instead of directly subtracting the coordinates of one point from another, here the distance is calculated by considering the earth as a sphere, and the distance between two coordinates is the length of the arc with the earth’s radius as the radius of the arc (R). One end of the arc is at the center of the ecosystem (lat1, lon1), and the other end (lat2, lon2) is the point to which distance needs to be calculated.
**Algorithm 5:** Haversine Distance Calculation **Start**;
 **Function** haversine(lat2, lon2):
  **Define** fixed coordinates:
   lat1 = 37.77492851, lon1 = 37.77491636
▹ Fixed reference point   R = 6371 ×$10^{3}$

▹ Earth’s radius in meters  **Convert** latitude and longitude values from degrees to radians:
   lat1_rad = radians(lat1)
   lon1_rad = radians(lon1)
   lat2_rad = radians(lat2)
   lon2_rad = radians(lon2)
  **Calculate** differences between the coordinates:
   dlon = lon2_rad - lon1_rad
   dlat = lat2_rad - lat1_rad
  **Apply** the Haversine formula:
   a = sin(dlat/2)^2^ + $cos\left(lat1\_rad\right)\times cos\left(lat2\_rad\right)\times sin{\left(dlon/2\right)}^{2}$
   c = 2×arcsin(sqrt(a))
  **Return** the distance: c × R


#### 4.2.7. Orchestrator Model

The Orchestrator model controls all operations in the ecosystem. The Orchestrator coordinator block controls and coordinates the operations of different blocks inside the Orchestrator. For the simulation proposed, the number of customers and the preferred mode of ecosystem operation needs to be input into the system. Based on the number of customers, the customer data required is generated by the simulation of the customer data block. The sensor data block generates the sensor metadata, which are GPS coordinates of the sensors and acceleration of the sensors for each time stamp. The GPS coordinates are generated using the Haversine formula as shown in Algorithm 5, from which the acceleration is calculated. After calculating the scores for different modes, the mode of operation (priority) is chosen and the data collection rates are subsequently calculated. To check the presence of an emergency condition, the vehicle’s velocity is calculated from its coordinates, and upon detection of a vehicle moving at a speed above the threshold speed, the proximity checker block finds the proximity to human beings in the environment. The Haversine calculator is the block used to find out the distance between two GPS coordinates. Simulation of the sensor battery values in accordance with the data collection rate is carried out in the sensor battery value calculation block. The cost associated with data collection for each customer is also calculated by the Orchestrator. The computational limits of the proposed model have been evaluated through simulations involving up to 20,000 devices. The results demonstrate that the model can efficiently handle this scale of devices using an Intel i7 processor with 32 GB RAM. The system’s architecture is designed to accommodate a large number of devices, with computational limitations primarily dependent on the underlying hardware configuration.

## 5. Results Discussion

**Figure 4:**
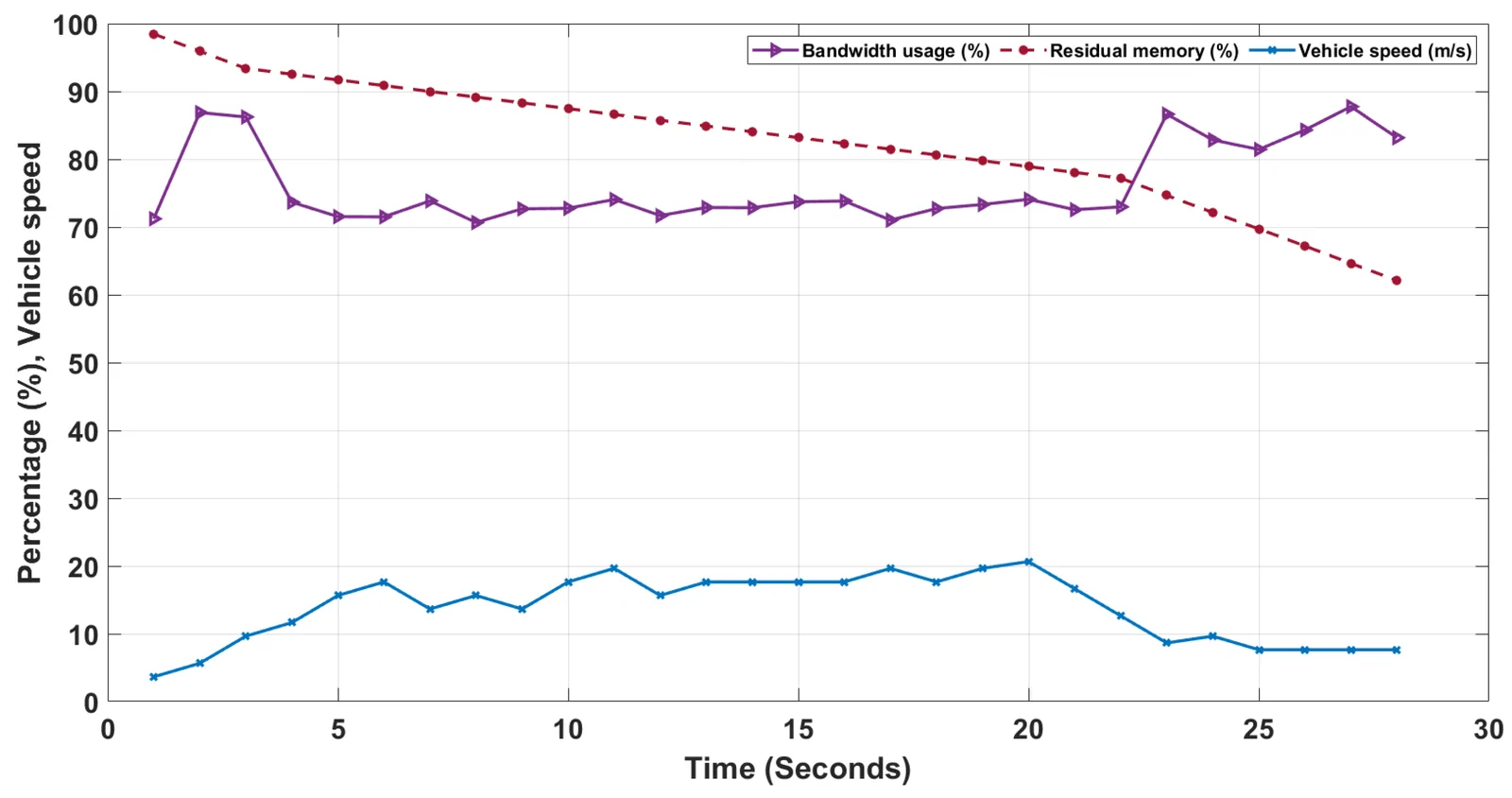
BW Consumption and Residual Memory.

The ecosystem works in its preferred condition until an emergency situation occurs. As the vehicle enters the ecosystem and moves at a speed above the limits, an emergency condition arises. As shown in Figure 4, the vehicle’s speed is within the speed limit (10 m/s) until timestamp 3. As soon as it crosses the speed limit, the BW usage dips from around 2.2%/s to 0.86%/s and stays in this range until the vehicle comes within the speed limit, which happens at timestamp 3, where velocity is 8.65 m/s. This is mainly because of the emergency mode activation, during which the data collection focus is on the data relevant to the emergency condition, such as frame collection. In this situation, the allocation of available BW for frame collection is high. Similarly, in the case of memory usage, the rate of memory consumption is high in the absence of emergency conditions. As soon as the emergency condition emerges, the memory consumption slows down and returns to the normal rate once the vehicle comes to the speed limit. The dip in the memory consumption rate is due to the decrease in the collection of metadata that are irrelevant in the emergency condition.

**Figure 5:**
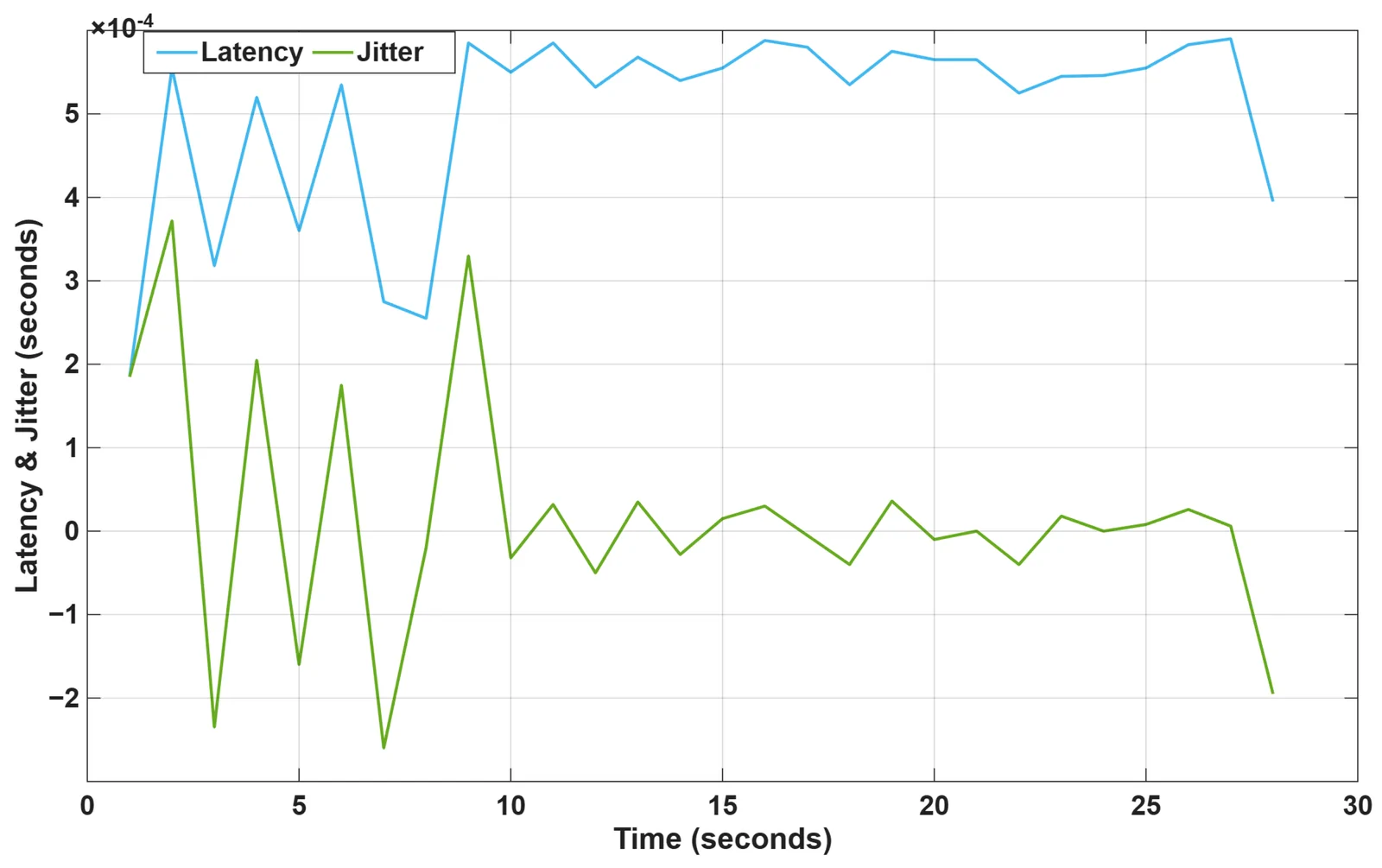
Latency and Jitter rate.

**Figure 6:**
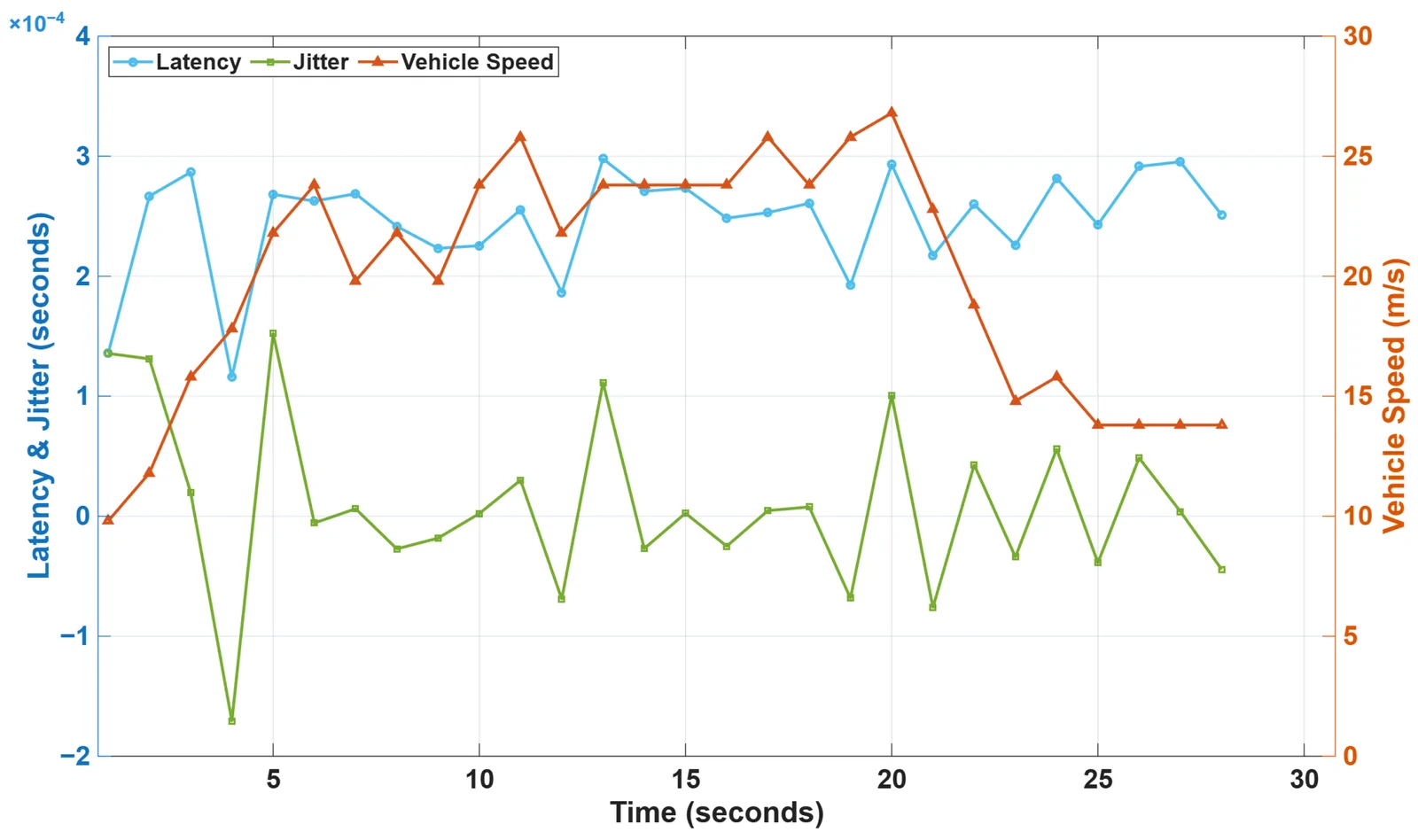
Latency Jitter rate and vehicle speed from an another simulation.

**Figure 7:**
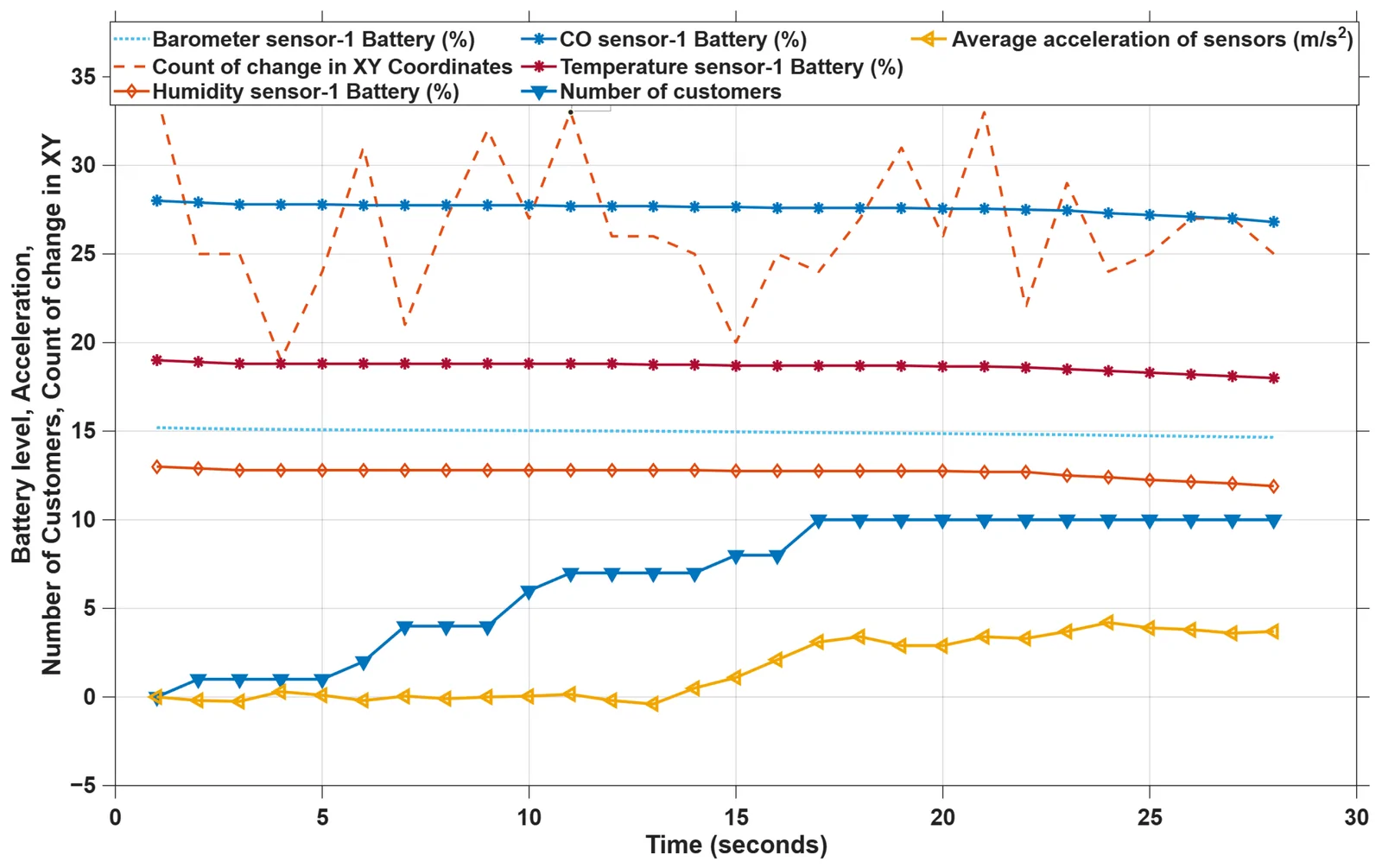
Battery Consumption and Number of Customers.

Similarly, the emergency condition affects the jitter and latency, as shown in Figure 5. The latency increases and stays almost constant in the emergency mode as the system fully focuses on the emergency condition. Figure 6 shows the jitter, latency and vehicle speed relation from another simulation, in which the emergency condition arises at the 2nd time stamp, and from there onwards the latency stays without much fluctuation. The battery levels of different sensors are plotted in Figure 7.

Higher data collection rates require more frequent data transmission, which consumes more battery power. Since the rate of battery consumption is directly proportional to the data collection of the sensor, with the decrease in the sensor data collection rate in emergency mode, the battery consumption becomes low, and it comes back to the normal rate at timestamp 23 s, where the ecosystem goes to normal mode operation. The average temperature sensor’s battery consumption is shown in Figure 7, where the average battery consumption rate decreases from 0.11%/s to 0.01%/s. The graph also shows the number of customers present in the environment at a given timestamp based on their location, whether it is in the boundary or not. Since GPS coordinates for the customers are simulated in a way in which the customers are coming into the environment from different distances, the number of customers keeps increasing and becomes stable after a certain period of time. At timestamp 1, the number of customers present in the environment is three. As time goes by, it increases and reaches the maximum number of customers of ten by time stamp 17. The count of the number of changes in the GPS coordinates of sensors and the average acceleration of the sensors for each second is also shown.

**Figure 8:**
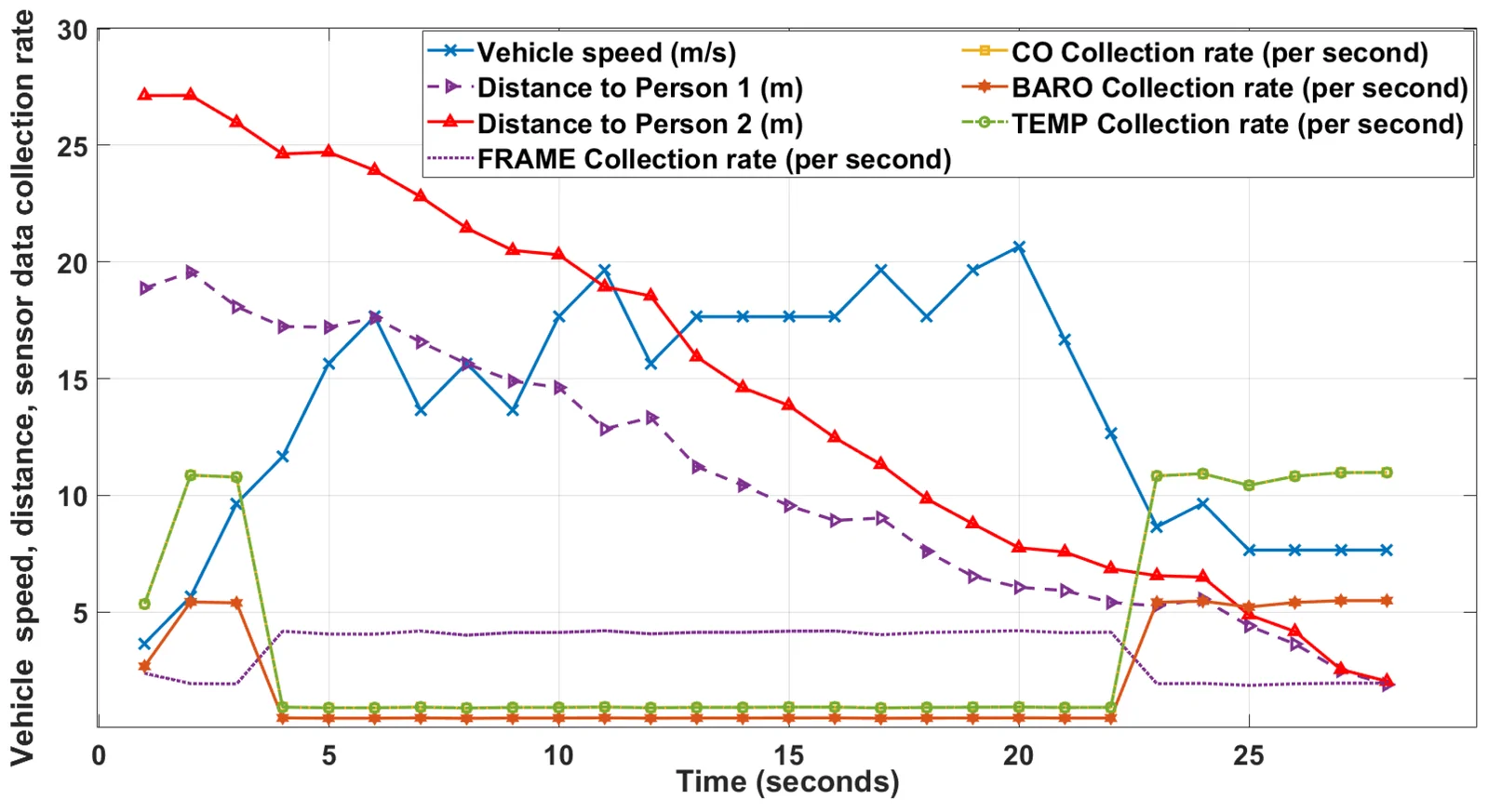
Impact of Emergency Situation on Data Collection Rate.

**Figure 9:**
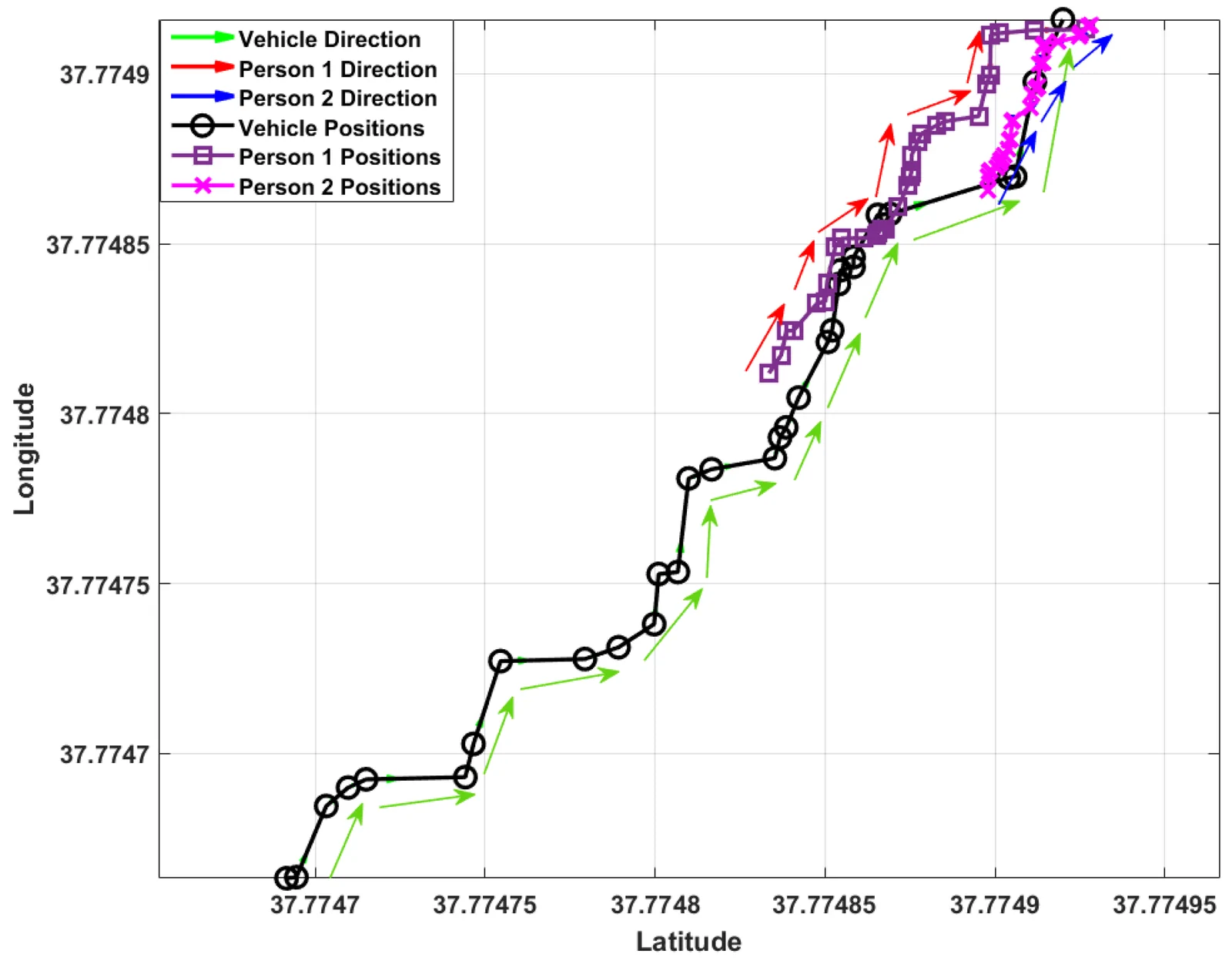
Vehicle and People Movement in Emergency Mode Scenario.

The graph in Figure 8 shows the effect of the emergency condition on different metadata collections. The sensor data collections, such as CO, temperature, barometer and humidity, which are less relevant in the emergency mode, decrease and the frame collection becomes high. This can be seen from time-stamp 4 to timestamp 23 s. The collection rate is different for different sensors. In the case of temperature sensors, the average collection rate changes from the range of 10 times/s to 1 time/s. The proximity of the speeding-up vehicle to the person present in the vehicle speeding-up scenario is also shown in Figure 8. Since the GPS coordinates of the customers and vehicle in this scenario are generated in such a way that all of them are moving towards the center of the environment, the proximity of both customers gets decreased as the time goes by. This has been plotted in Figure 9. The GPS coordinates of both customers and the vehicle for 28 s with their direction of movement are shown. During emergency situations, SLAs for critical services are prioritized, while non-critical services are adjusted to maintain minimum acceptable levels. This ensures that essential services remain operational, albeit with potential degradation of non-critical services. When compared with state-of-the-art solutions in Table 1, the proposed model shows clear advantages for IoMT-based environments. It not only provides high scalability and low latency but also ensures adaptability and SLA maintenance during emergency conditions, where most existing solutions degrade.

## 6. Conclusions, Limitations and Future Research

The proposed orchestration mechanism optimizes data collection by driving the ecosystem into the required mode, assessing real-time conditions, and ensuring efficient resource allocation. By comparing the optimized metadata collection rate with the minimum acceptable data collection rate as per the SLAs of live customers, the proposed model addresses potential SLA breaches. Different modes of operation enable the environment to work from the perspective of various stakeholders. Furthermore, this research study demonstrates the potential impact of emergency conditions on ecosystem operation and how efficiently the proposed model handles such situations. Since the proposed model exhibits key performance indicators of an ecosystem, such as flexibility, efficient resource allocation, avoidance of SLA breaches, and the ability to handle a large number of customers, it proves to be more efficient. However, despite promising outcomes, the addition of a number of edge servers for the efficient allocation of BW and memory usage and the approach to dynamic allocation of available BW for meta data collection could provide better results. The current work is limited to a simulated environment and does not include deployment or validation on real cloud-native or containerized platforms. Future research should focus on extending the Orchestrator into scalable, multi-cloud, and microservice-based implementations to better assess performance and adaptability in real-world scenarios. This approach can also be used to further compare the Orchestrator’s performance against state-of-the-art deployments. In addition, the current simulation does not fully capture the network variability and uncertainties of a real port environment, such as network fluctuations, sensor or device failures, noisy or missing information, and temporary connectivity issues. The proposed orchestrator also assumes the availability of the information required for decision-making, representing an idealized information environment. Furthermore, although metadata are anonymized during normal operation, emergency-mode de-anonymization introduces a privacy trade-off that requires appropriate authorization, access control, audit logging, retention policies, and compliance with applicable data-protection requirements in a real deployment. These privacy controls are not implemented in the current simulation and therefore represent an additional limitation of the proposed approach. Future work will investigate privacy-aware access control and selective disclosure mechanisms to ensure that only the minimum information required for emergency response is revealed. Overall, this paper stands as a comprehensive guide for the implementation of an IoT orchestrator engine.

## Figures and Tables

**Table 1:** Comparative analysis of orchestration frameworks.

Framework	Domain Focus	Scalability	Latency/ Responsiveness	Resilience & Adaptability	Evaluation Mode
SliceSphere [10]	Cloud-native application slicing	Very high (multi-cloud, multi-tenant slicing via “SliceKube”)	Moderate—optimised for infrastructure orchestration, not per-event latency	High; provides slice isolation and control-plane recovery	Testbed (SAVI 2.0)
Big Data Apps Composition Environment (BDCE) [9]	Big-data workflow orchestration	High (multiple executors, scalable microservices)	Medium; not real-time focused	Medium; resilience via workflow re-execution	Prototype + GUI simulation
Container-based IoT Management [8]	IoT device orchestration (GCP + MQTT + Kubernetes)	Medium; limited by static container pool	Low orchestration latency in steady state	Moderate; manual scaling	Cloud deployment (GCP GKE)
Proposed IoMT Orchestrator	Mobile IoT/Smart Port ecosystems	High; dynamic scaling via simulated resource pools	Low latency under normal load; maintains SLA under mobility	High; adapts under emergency/variable customer load	Simulation

**Table 2:** Notation used in the cost calculation algorithm.

Symbol	Description
$C_{r,T}$	Temperature collection rate
$C_{r,B}$	Barometric pressure collection rate
$C_{r,H}$	Humidity collection rate
$C_{r,CO}$	Carbon monoxide collection rate
$C_{r,M}$	Memory data collection rate
$f_{r}$	Frame collection rate
$D_{s,T},D_{s,B},D_{s,H}$	Data sizes for temperature, barometric pressure, and humidity
$D_{s,CO},D_{s,M},D_{s,F}$	Data sizes for carbon monoxide, memory, and frames
$C_{c,T},C_{c,B},C_{c,H}$	Collection costs for temperature, barometric pressure, and humidity
$C_{c,CO},C_{c,M},C_{c,F}$	Collection costs for carbon monoxide, memory, and frames
$C_{T},C_{B},C_{H},C_{CO},C_{M},C_{F}$	Cost per second for each respective attribute
*P*	Subscription priority
$C_{\mathrm{total}}$	Total cost per second

## Data Availability

The data that support the findings of this study are available from the corresponding author, CP, upon reasonable request.
